# Bilateral dentigerous cyst in a non-syndromic patient: Report of an unusual case with review of the literature

**DOI:** 10.4103/0973-029X.80017

**Published:** 2011

**Authors:** Avinash Tamgadge, Sandhya Tamgadge, Daivat Bhatt, Sudhir Bhalerao, Treville Pereira, Mukul Padhye

**Affiliations:** *Department of Oral Pathology and Microbiology, Dr D.Y. Patil Dental College and Hospital, Nerul, Navi Mumbai, India*

**Keywords:** Bilateral dentigerous cyst, non-syndromic, syndromic

## Abstract

Dentigerous cysts are the most common developmental cysts of the jaws, most frequently associated with impacted mandibular third molar teeth and impacted canines. Bilateral dentigerous cysts are rare and occur typically in association with a developmental syndrome. The occurrence of bilateral dentigerous cysts is rare and, to date, only 21 cases have been reported in literature till 2009. Here, we report a case of bilateral dentigerous cysts in maxillae of non-syndromic 10-year-old patient with brief review of literature.

## INTRODUCTION

A dentigerous cyst is an epithelial-lined developmental cavity that encloses the crown of an unerupted tooth at the cementoenamel junction.[1] Dentigerous cysts are the second most common odontogenic cysts after radicular cysts, accounting for approximately 24% of all true cysts in the jaws. Their frequency in the general population has been estimated at 1.44 cysts for every 100 unerupted teeth.[2] The cyst arises from the separation of the follicle from the crown of an unerupted tooth, and although it may involve any tooth, the mandibular third molars are the most commonly affected. Dentigerous cysts are frequently discovered when radiographs are taken to investigate a failure of tooth eruption, a missing tooth or malalignment.[3] There is usually no pain or discomfort associated with the cyst unless it becomes secondarily infected. Radiographs show a unilocular, radiolucent lesion characterized by well-defined sclerotic margins and associated with the crown of an unerupted tooth. While a normal follicular space is 3-4 mm, a dentigerous cyst can be suspected when the space is more than 5 mm.[4] Most dentigerous cysts are solitary.[4] Bilateral and multiple cysts are usually found in association with a number of syndromes including cleidocranial dysplasia, Maroteaux-Lamy syndrome[2] and in mucopolysaccharidosis.[5] In the absence of these syndromes, bilateral dentigerous are rare. Here, we report the unusual occurrence of non-syndromic bilateral dentigerous cysts associated with right maxillary second premolar and left maxillary canine.

## CASE REPORT

**Figure 1:**
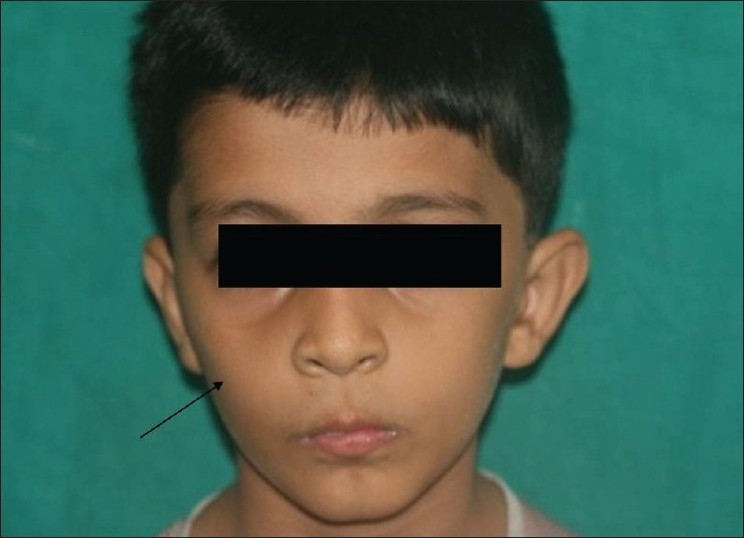
Clinically shows asymmetry on right side of face

**Figure 2:**
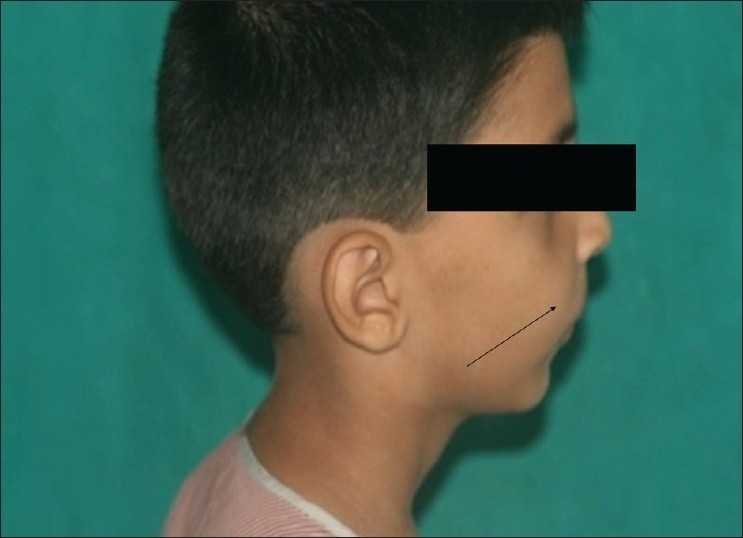
Right profile shows definite swelling

**Figure 3:**
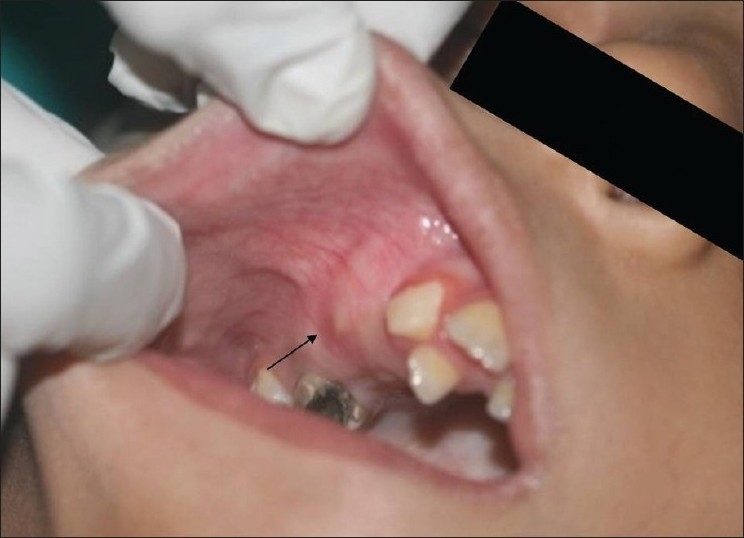
Intraorally right side shows swelling in 14 and 15

**Figure 4:**
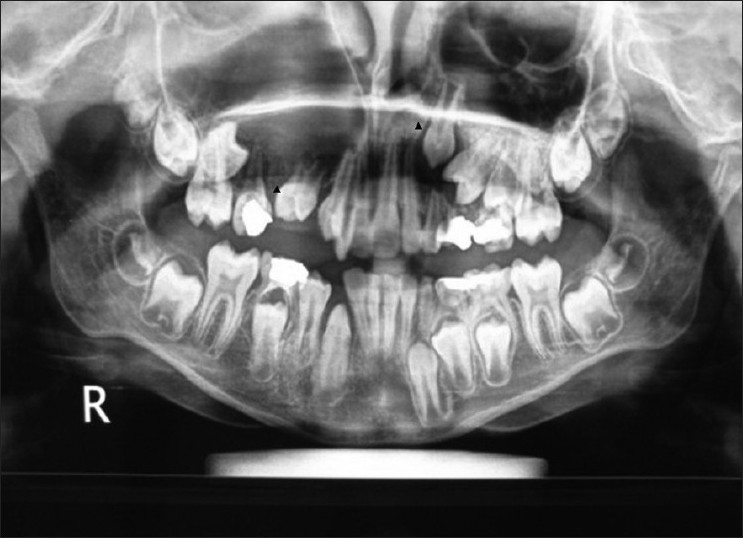
OPG after 1 year showing definite increase in radiolucency with 15 and 23. Follicular space more than 5 mm related to 23

**Figure 5:**
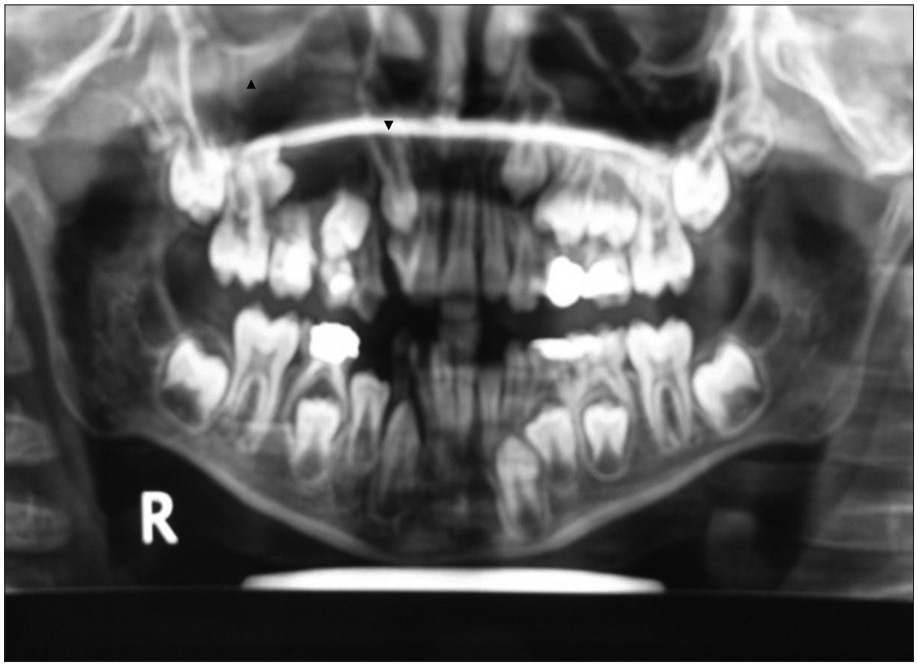
OPG taken 1 year before shows follicular space with 23 and radiolucency with 15

**Figure 6:**
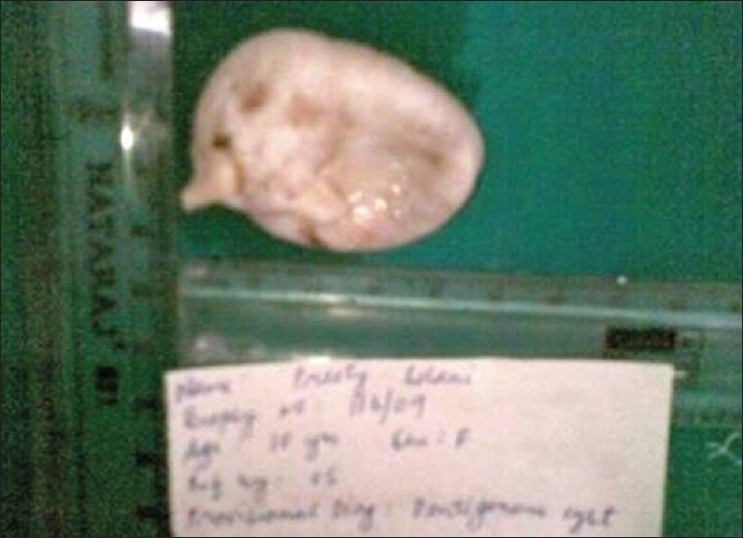
Gross pathology of right side

**Figure 7:**
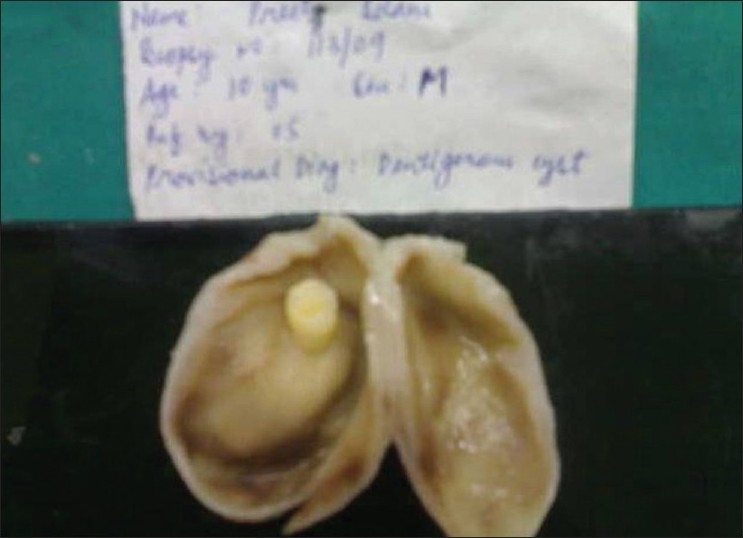
Cut section of the cyst showing impacted 15

**Figure 8:**
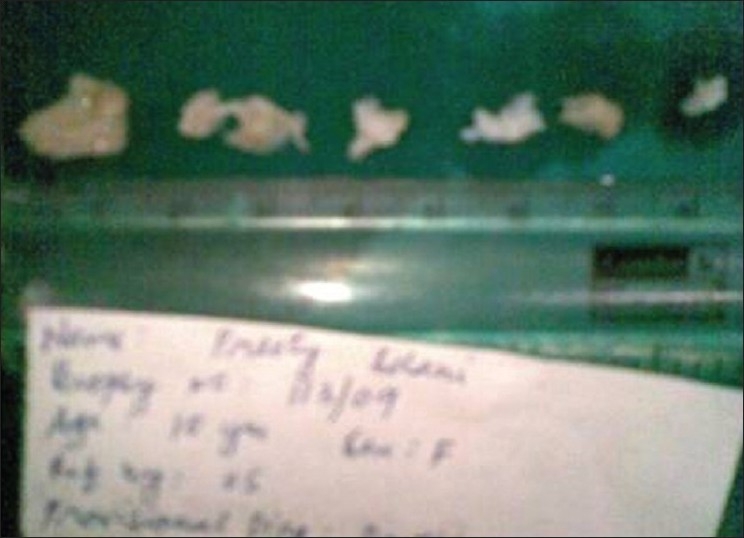
Gross pathology of left side showing multiple bits of tissue

A 10-year-old boy was referred to the department of oral surgery at Dr D.Y. Patil Dental College and Hospital for the evaluation of an asymptomatic, cystic lesion in the right maxilla [Figures 1 and 2]. Intraoral examination [Figure 3] revealed a mixed dentition and clinically absent premolars and there was definite swelling in association with unerupted premolars i.e., 14 and 15. Slight extraoral swelling or tenderness in relation to the maxilla on same side was noted [Figure 3]. The patient’s medical history was non-significant and no associated syndromes were present. On routine radiographical examination it was found that there was follicular enlargement on the contralateral side of maxilla in relation to permanent canine [Figure 4]. On examination of an old radiograph taken 1 year back there was definite follicular enlargement in same area [Figure 5]. A panaromic radiograph showed thin sclerotic border surrounding the well-defined unilocular radiolucent area that was present on right side of maxilla in relation to second premolar and similar sclerotic margin was evident even on left side of maxilla in relation to permanent canine. The contents of the swelling were aspirated and sent for investigations, the result of which was consistent with the diagnosis of a infected cystic lesion. After clinical and radiological examination, a provisional diagnosis of bilateral dentigerous cyst was made; however, large periapical cyst, odontogenic keratocyst, adenomatoid odontogenic tumor and ameloblastic fibroma were also considered in the differential diagnosis. Routine blood and urine examination was advised; the results were within normal limits. Surgical enucleation of the cyst was chosen as the treatment of choice. The surgical specimens were then sent to department of Oral and Maxillofacial Pathology and Microbiology for final diagnosis [Figures 6, 7 and 8].

## FNAC REPORT

FNAC of right side showed cholesterol crystals with inflammatory cells.

## HISTOPATHOLOGICAL FINDINGS

**Figure 9:**
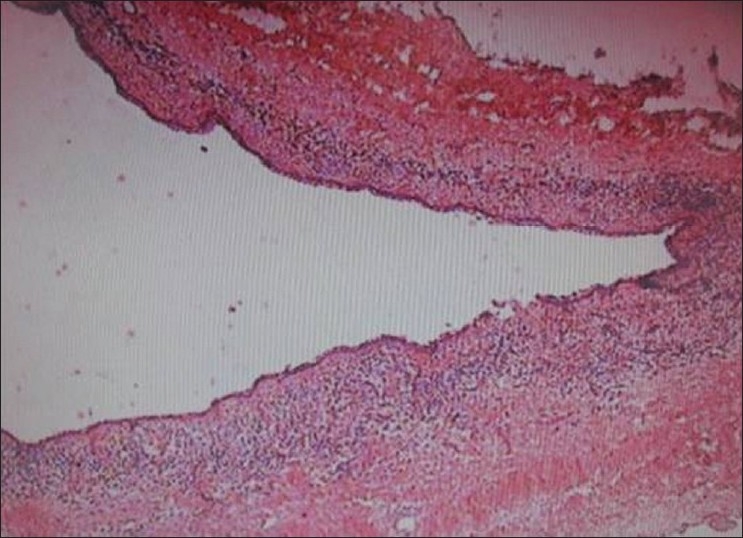
Photomicrograph of the lesion shows features suggestive of dentigerous cyst. (H and E, original magnification 40×) right side

**Figure 10:**
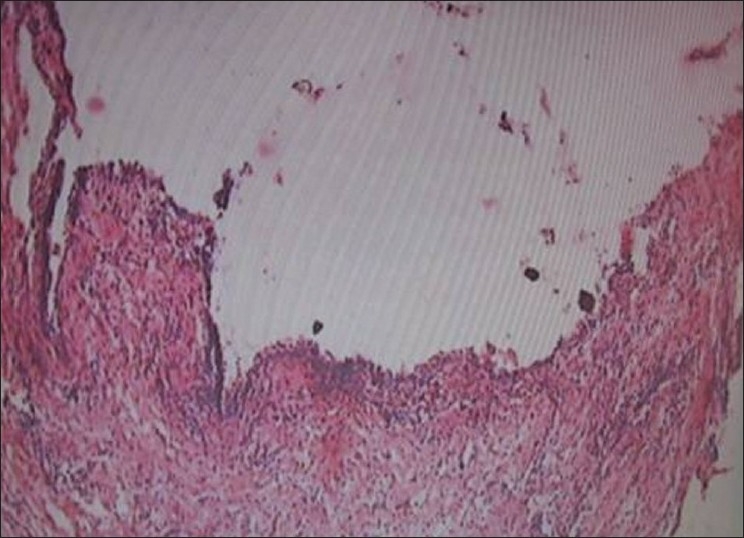
Photomicrograph of the lesion shows features suggestive of dentigerous cyst. (H and E, original magnification 40×) left side

**Figure 11:**
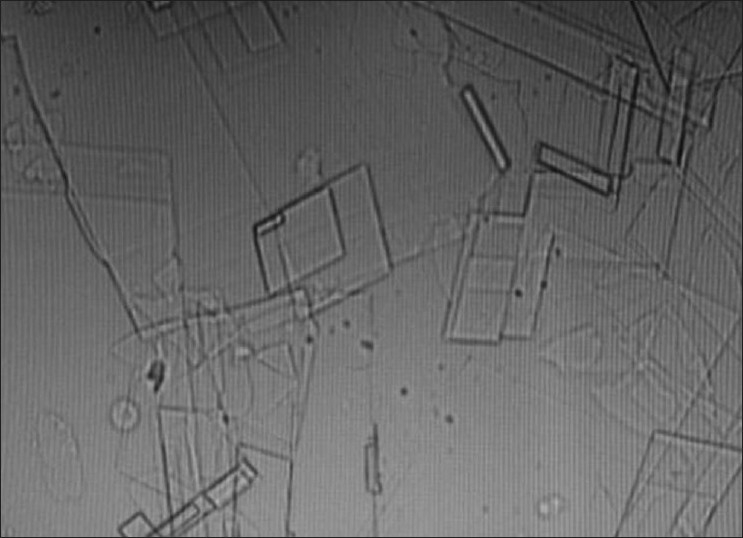
FNAC of right side showing cholesterol crystals

Histologically both the specimens were similar and showed a thin fibrous cystic wall lined by a 2-3 cell layers thick non-keratinized stratified squamous epithelium. Retepegs were absent and the connective tissue showed inflammatory cell infiltrate. Subepithelial layers showed parallel bundles of collagen fibers observed at periphery. These findings confirmed diagnosis of dentigerous cysts [Figures 9, 10 and 11].

## DISCUSSION

A dentigerous cyst can be defined as a cyst that encloses the crown of an unerupted tooth, expands the follicle and is attached to the cementoenamel junction of the unerupted tooth. The substantial majority of dentigerous cysts involve the mandibular third molar and the maxillary permanent canine, followed by the mandibular premolars, maxillary third molars and rarely the maxillary premolars. Studies have shown that the incidence rate of dentigerous cysts involving the maxillary premolar was 2.7% as compared to 45.7% involving the mandibular third molar. Mourshed stated that 1.44% of impacted teeth undergo dentigerous cyst transformation, so dentigerous cysts involving the premolars are rare. Daley *et al*, reported an incidence rate of 0.1-0.6%, whereas Shear found the incidence to be 1.5%.[6] Dentigerous cysts most commonly occur in the 2^nd^and 3^rd^decades of life.[6]

These lesions can also be found in children and adolescents and show a male predilection.[2,7] In the present case report, the dentigerous cyst was associated with the maxillary right second premolar and left canine in a 10-year-old male child.

The exact histogenesis of the dentigerous cyst is not known. It is stated that the dentigerous cyst develops around the crown of an unerupted tooth by accumulation of fluid either between the reduced enamel epithelium and enamel or in between the layers of the enamel organ.[4] This fluid accumulation occurs as a result of the pressure exerted by an erupting tooth on an impacted follicle, which obstructs the venous outflow and thereby induces rapid transudation of serum across the capillary wall. Toller stated that the likely origin of the dentigerous cyst is the breakdown of proliferating cells of the follicle after impeded eruption. These breakdown products result in increased osmotic tension and hence cyst formation. Bloch suggested that the origin of the dentigerous cyst is from the overlying necrotic deciduous tooth. The resultant periapical inflammation will spread to involve the follicle of the unerupted permanent successor; an inflammatory exudate ensues and results in dentigerous cyst formation.[3] Most of the authors have reported the presence of carious or discolored deciduous teeth in relation to the development of dentigerous cysts. This suggests that the periapical inflammatory exudates from the deciduous teeth might be one of the risk factor for the occurrence of dentigerous cysts.[3]

Although dentigerous cysts are common developmental cysts, bilateral dentigerous cysts are extremely rare and hardly reported. Bilateral or multiple dentigerous cysts are usually associated with the Maroteaux-Lamy syndrome, mucopolysaccharidosis type VI), cleidocranial dysplasia, Basal cell nevus syndrome[8] and may sometimes are suggested to be induced by prescribed drugs. The combined effect of cyclosporin and a calcium blocker is reported to cause bilateral dentigerous cyst.[9] Pleomorphism in chromosome 1qh+ has also been reported with this condition.[10] In our case, there were no clinically evident syndromes.

Bilateral dentigerous cysts are rare in the absence of an underlying syndrome or systemic disease. An extensive search of the English language literature has identified only 21 cases [Table 1].[5,9,11,12] Although this finding may reflect the true rarity of the condition, it is conceivable that bilateral dentigerous cysts are either under recognized or under-reported as sometimes they are known to regress spontaneously.[13]

**Table 1:** Table showing review of literature of bilateral dentigerous cyst

S. no.	Athors/Year	Sex	Age (years)	Location	Treatment
1	Myers, 1943	F	19	Md. third molars	Enucleation
2.	Henefer, 1964	F	52	Mx. third molars	Enucleation
3.	Stanback, 1970	M	9	Md. first molars	Enucleation
4.	Callaghan, 1973	M	38	Md. third molars	Enucleation
5.	Burton, Scheffer, 1980	F	57	Md. third molars	Enucleation
6.	Swerdloff, Alexander, Ceen, Ferguson, 1980	F	7	Md. first molars	Enucleation
7.	Crinzi, 1982	F	15	Md. third molars	Enucleation
8.	McDonnell, 1988	M	15	Md. second premolar and second molar	Enucleation
9.	Eidinger, 1989	M	15	Md. first molars	Enucleation
10.	O’Neil, Mosby, Lowe, 1989	M	5	Md. first molars	Enucleation
11.	Banderas, Gonzalez, Ramirez, Arroyo, 1996	M	38	Md. third molars	Enucleation
12.	Sands, Tocchio, 1998	F	3	Md. central incisors and first molars	Enucleation
13.	Ko, Dover, Jordan, 1999	M	42	Md. third molars	Enucleation
14.	De Biase, Ottolenghi, Polimeni, Benvenuto, Lubrano, Magliocca, 2001	M	8	Md. first molars	Enucleation
15.	Shah, Thuau, Beale, 2002	M	39	Md. third molars	No treatment
16.	Ustuner, Fitoz, Atasoy, Erden, Akyar, 2003	M	6	Mx. Canines	Enucleation
17.	Batra, Roychoudhury, Balakrishan, Parkash, 2004	F	15	Md. third molars and second premolar	Enucleation
18.	DQ Frietas, *et al*[5]	M	13	Mx third molars and Md second molar	Enucleation
19.	Turkiye[9]	M	51	Md third molars	Enucleation
20.	Fregnani ER, *et al*,[11]	M	5	Md first molars	Enucleation
21.	Sergio EV. *et al*,[12]	M	5	Md molars	Enucleation

M, male; F, female; Md., mandibular; Mx., maxillary

The age range for the reported cases varies widely, from 5 to 57 years of age. Eight of the cysts occurred in children under the age of 12 as with our case. All but three cases 6, 12, 13 were identified at ages corresponding to the normal eruption times of the affected teeth. As with our case, these three cases occurred in asymptomatic individuals, which accounts for the delayed diagnosis. All but three of the 21 cases 2, 16, 18 have been associated with mandibular molar teeth. But bilateral dentigerous cyst s affecting premolar and canine are rare as with our case. There have been no reported cases of non-syndromic, bilateral dentigerous cysts occurring in all four dental quadrants. Since cysts can attain considerable size with minimal or no symptoms, early detection and removal of the cysts is important to reduce morbidity. Moreover, all but three of 21 reported cases, presented without pain. And, all but one was discovered during investigation of asymptomatic slow growing swellings. It is therefore important to perform radiographical examination of all unerupted teeth. While bite-wing and periapical radiography is typically performed in the routine examination of patients with a healthy dentition, this series of radiographs may occasionally fail to delineate the full extent of a lesion if present. A panoramic radiograph supplemented with skull series or more advanced imaging such as tomography may permit a better delineation of the extent of the lesion and its relationship to adjacent anatomical structures.[13]

Removal of associated tooth and enucleation of soft tissue component is definative therapy in most instances.[14] In case of maxillary cuspid teeth, the cyst may be excised or marsupialized and the tooth is brought into proper alignment in arch with aid of orthodontic appliance.[15]

## CONCLUSIONS

Bilateral dentigerous cyst is a very rare entity. In case of multiple dentigerous cyst, a through clinical and systematic examination should be done to rule out any associated syndrome.
